# In vivo efficacy of XR9051, a potent modulator of P-glycoprotein mediated multidrug resistance

**DOI:** 10.1038/sj.bjc.6690267

**Published:** 1999-04

**Authors:** P Mistry, J Plumb, S Eccles, S Watson, I Dale, H Ryder, G Box, P Charlton, D Templeton, P B Bevan

**Affiliations:** Xenova Ltd, 240 Bath Road, Slough, SL1 4EF, UK; CRC Department of Oncology, University of Glasgow, Glasgow, G61 1BD, UK; Institute of Cancer Research, Sutton, SM2 5NG, UK; Cancer Studies Unit, University of Nottingham, Nottingham, NG7 2UH, UK

**Keywords:** multidrug resistance, P-glycoprotein, XR9051, resistance modulators

## Abstract

Overexpression of P-glycoprotein (P-gp) is a potential cause of multidrug resistance (MDR) in tumours. We have previously reported that XR9051 (*N*-(4-(2-(6,7-dimethoxy-1,2,3,4-tetrahydro-2-isoquinolyl)ethyl)phenyl)-3-((3Z,6Z)-6-benzylidene-1-methyl-2,5-dioxo-3-piperazinylidene)methylbenzamide) is a potent and specific inhibitor of P-gp, which reverses drug resistance in several murine and human MDR cell lines. In this study we have evaluated the in vivo efficacy of this novel modulator in a panel of murine and human tumour models and examined its pharmacokinetic profile. Efficacy studies in mice bearing MDR syngeneic tumours (P388/DX Johnson, MC26) or human tumour xenografts (A2780AD, CH1/DOXr, H69/LX) demonstrated that co-administration of XR9051 significantly potentiated the anti-tumour activity of a range of cytotoxic drugs. This modulatory activity was observed following parenteral and oral co-administration of XR9051. In addition, the combination schedules were well-tolerated. Following intravenous administration in mice, XR9051 is rapidly distributed and accumulates in tumours and other tissues. In addition, the compound is well-absorbed after oral administration. These data suggest that XR9051 has the potential for reversing clinical MDR mediated by P-glycoprotien. © 1999 Cancer Research Campaign


*British Journal of Cancer* (1999) **79**(11/12), 1672–1678
© 1999 Cancer Research Campaign
Article no. bjoc.1998.0267

### In vivo efficacy of XR9051, a potent modulator of P-glycoprotein mediated multidrug resistance

**P Mistry[1], J Plumb[2], S Eccles[3], S Watson[4], I Dale[1], H Ryder[1], G Box[3], P Charlton[1], D Templeton[1] and PB Bevan[1]**

[1]Xenova Ltd, 240 Bath Road, Slough, SL1 4EF, UK; [2]CRC Department of Oncology, University of Glasgow, Glasgow, G61 1BD, UK; [3]Institute of Cancer Research, Sutton, SM2 5NG, UK; [4]Cancer Studies Unit, University of Nottingham, Nottingham, NG7 2UH, UK

**Summary** Overexpression of P-glycoprotein (P-gp) is a potential cause of multidrug resistance (MDR) in tumours. We have previously reported that XR9051 (*N*-(4-(2-(6,7-dimethoxy-1,2,3,4-tetrahydro-2-isoquinolyl)ethyl)phenyl)-3-((3Z,6Z)-6-benzylidene-1-methyl-2,5-dioxo-3-piperazinylidene)methylbenzamide) is a potent and specific inhibitor of P-gp, which reverses drug resistance in several murine and human MDR cell lines. In this study we have evaluated the in vivo efficacy of this novel modulator in a panel of murine and human tumour models and examined its pharmacokinetic profile. Efficacy studies in mice bearing MDR syngeneic tumours (P388/DX Johnson, MC26) or human tumour xenografts (A2780AD, CH1/DOXr, H69/LX) demonstrated that co-administration of XR9051 significantly potentiated the anti-tumour activity of a range of cytotoxic drugs. This modulatory activity was observed following parenteral and oral co-administration of XR9051. In addition, the combination schedules were well-tolerated. Following intravenous administration in mice, XR9051 is rapidly distributed and accumulates in tumours and other tissues. In addition, the compound is well-absorbed after oral administration. These data suggest that XR9051 has the potential for reversing clinical MDR mediated by P-glycoprotien.

**Keywords:** multidrug resistance; P-glycoprotein; XR9051; resistance modulators

Multidrug resistance (MDR) is known to compromise treatment of cancers with several structurally and functionally unrelated anticancer drugs, including anthracyclines, vinca alkaloids, epipodophyllotoxins, Paclitaxel, colchicine and actinomycin D. Although there are several different mechanisms associated with development of MDR, a common cause is believed to be over-expression of a plasma membrane P-glycoprotein (P-gp). This 170 kDa protein is encoded by the MDR1 gene and acts as an energy-dependent drug efflux pump, preventing adequate intracellular accumulation of cytotoxic drugs (for reviews see Gottesman and Pastan, 1993, Childs and Ling, 1994; Germann, 1996).

The first report of the pharmacological reversal of MDR was made by Tsuruo and colleagues in 1981 (Tsuruo et al, 1981), who showed that the calcium channel blocker verapamil and the calmodulin antagonist trifluoperazine were able to potentiate the cytotoxic effect of vincristine and produce an increased cellular accumulation of vincristine in a murine MDR cell line. A large number of lipophilic, positively charged compounds have subsequently been identified as MDR modulators, which function by blocking P-gp-mediated drug efflux. The initial MDR modulators, such as verapamil, cyclosporin A, FK506, quinine, trifluoperazine and tamoxifen, were originally developed for pharmacological uses other than inhibition of MDR. For many of these drugs, widespread clinical use as MDR modulators has been precluded because of side-effects associated with their use at concentrations required to inhibit P-gp (Lum et al, 1993; Ferry et al, 1996). However, some of these modulators, such as verapamil and cyclosporin A, have shown clinical benefit in the treatment of refractory haematopoietic malignancies (Dalton, 1994). Hence the development of a more specific and potent modulator of MDR seemed desirable. Indeed, recently, some potent second-generation modulators have been developed and the most potent of these include PSC 833 (Boesch et al, 1991), GF120918 (GG918) (Hyafil et al, 1993) and LY335979 (Dantzig et al, 1996). Some of these compounds are currently in clinical trials.

XR9051, a novel, potent and specific modulator of P-gp-mediated MDR, was derived from the chemical modification of a lead natural product diketopiperazine, isolated from a Streptomyces species. In vitro, co-exposure to 300–500 nM XR9051 fully reverses resistance to a range of cytotoxic drugs in several murine and human P-gp-expressing cell lines (Dale et al, 1998). The present studies were performed to evaluate the in vivo efficacy and pharmacokinetic profile of XR9051. The results demonstrate that XR9051 has a large volume of distribution, is well-absorbed orally and is a potent modulator of MDR in vivo. The modulatory activity was observed in several tumour models with a range of cytotoxic agents at well-tolerated doses. Hence XR9051 could potentially have significant clinical benefit in the treatment of MDR cancers.

### MATERIALS AND METHODS

### Materials

Epirubicin and doxorubicin were obtained from Pharmatalia (St Albans, UK). Paclitaxel and etoposide were purchased from Bristol-Myers Squibb Pharmaceuticals Ltd (Middlesex, UK). Vincristine was purchased from David Bull Laboratories (Warwick, UK). 1,2-Propanediol (propylene glycol) and D-(+)-glucose (dextrose) were obtained from Sigma UK Ltd. XR9051 was synthesized as a mesylate salt at Xenova Ltd and was dissolved in propylene glycol–5% dextrose (3/2, v/v) at 20 mg ml$^{-1}$

*Received 14 April 1998*
*Revised 27 July 1998*
*Accepted 7 August 1998*

*Correspondence to:* P Mistry and diluted appropriately in 5% dextrose immediately before use. All cytotoxic drugs were of clinical grade and were diluted to the desired concentration in 0.9% sodium chloride solution or 5% (w/v) dextrose solution. All solutions were kept in the dark.

### In vivo efficacy evaluation

### *P388 murine leukaemia model*

All animal experimentation was performed to Home Office regulations and the UKCCCR guidelines were adhered to throughout all anti-tumour studies. The P388/P-sensitive murine leukaemia cell line and the P388/DX Johnson drug-resistant subline, derived by continuous exposure to doxorubicin (Johnson et al, 1978) were provided by Dr M Grandi (Pharmacia Upjohn, Milan, Italy). In vitro both cell lines were maintained in RPMI-1640 medium supplemented with 10% fetal calf serum (FCS), 2 mM L-glutamine and 6 mM 2-mercaptoethanol at 37°C in an atmosphere of 95% air–5% $CO_2$. Drug-resistant cells were maintained under positive selective pressure by treatment with 0.25 μg ml$^{-1}$ doxorubicin for one passage out of every six to eight passages. These cells were subsequently incubated in drug-free medium for one passage before experimental use.

For in vivo studies cells were maintained by weekly intraperitoneal (i.p.) passage of $10^6$ cells in 6–8-week-old female (C57BL/6 X DBA/2) F1 hybrid mice. The cells underwent a minimum of two in vivo passages before use in efficacy studies and they were discarded after 20 passages. Preliminary studies showed that increasing the number of parental and resistant cells in the inoculum from $10^1$ to $10^5$ cells progressively shortened survival time of mice. Thereafter, no further decrease in survival time was observed with increase in cell inoculum up to $10^7$ cells. Hence for efficacy studies, female F1 hybrid mice were inoculated with $10^5$ P388/P or P388/DX Johnson cells i.p. in 1 ml of phosphate buffered saline (PBS). Following cell inoculation (day 0), the animals were randomized into groups of 8–12. The groups were treated 24 h after cell inoculation with various schedules of vincristine or doxorubicin with and without XR9051. Vincristine (0.1 or 0.2 mg kg$^{-1}$, in 10 ml kg$^{-1}$) was administered i.p. on days 1–5 and doxorubicin (2 mg kg$^{-1}$ in 10 ml kg$^{-1}$) was administered i.p. on days 1, 4 and 7 after cell inoculation. XR9051 was administered either i.p. at the same time as, or intravenously (i.v.) or orally (p.o.) 1 h before, the cytotoxic drugs. The animals were monitored daily and killed by cervical dislocation when they became moribund. Statistical evaluation of survival data was performed using the Kaplan–Meier log-rank test.

### *MC26 murine colon carcinoma model*

In vivo efficacy was also evaluated using MC26 murine colon carcinoma tumours that exhibit intrinsic drug resistance which is at least partly mediated by expression of P-gp (Spoelstra et al, 1991). The cell line, which grows in vitro as a loosely-attached monolayer, was maintained in RPMI-1640 medium supplemented with 10% FCS and 2 mM L-glutamine at 37°C in an atmosphere of 95% air–5% $CO_2$. The MC26 tumours were initially established by subcutaneous (s.c.) injection of $1 \times 10^5$ cells into female Balb/c mice. Once the tumours had reached a mean diameter of about 1 cm they were removed and minced, and a small portion of the tumour slurry implanted s.c. in the right flank of female Balb/c mice (age 8–12 weeks). The established tumours were similarly passaged a minimum of two times before use in efficacy studies. For the efficacy studies, tumour slurry was implanted (day 0) and

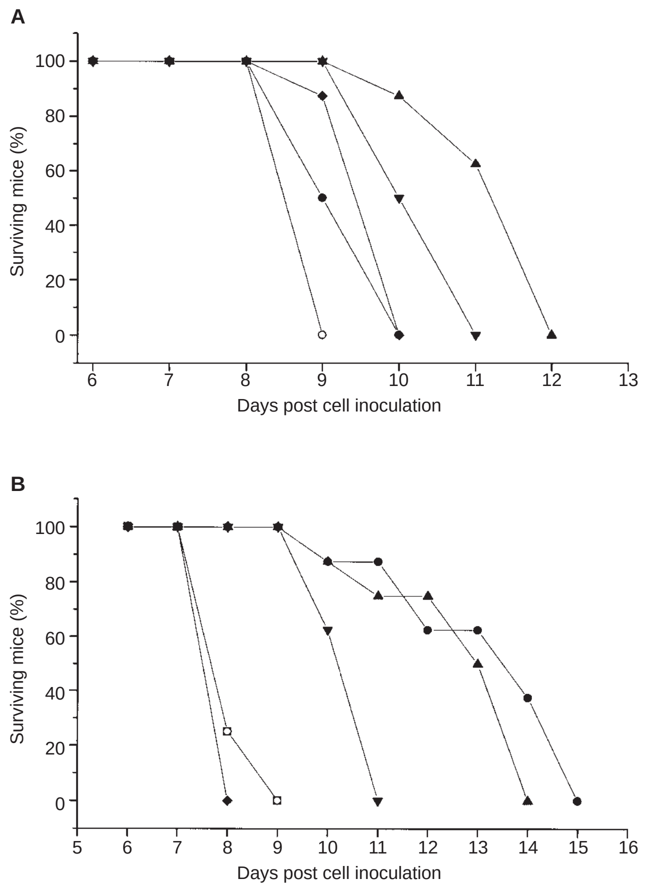

**Figure 1** Sensitization of vincristine anti-tumour effect on P388/DX Johnson murine leukaemia-bearing mice by XR9051 administered (**A**) i.v. and (**B**) p.o. P388/DXJ cells ($10^5$) were implanted i.p. and treatment was started 24 h later. The modulator was administered 1 h before the cytotoxic drug. (**A**) Tumour-bearing mice were treated on days 1–5 with vincristine 0.1 mg kg$^{-1}$ i.p. (○), XR9051 40 mg kg$^{-1}$ i.v. (●), or combination of vincristine with XR9051 i.v. at 40 mg kg$^{-1}$ (▲), 20 mg kg$^{-1}$ (▼) or 10 mg kg$^{-1}$ (◆). (**B**) Tumour-bearing mice were treated in days 1–5 with vincristine 0.1 mg kg$^{-1}$ i.p. (○), XR9051 80 mg kg$^{-1}$ i.v. (◆) and vincristine with XR9051 p.o. at 120 mg kg$^{-1}$ (●), 80 mg kg$^{-1}$ (▲) or 60 mg kg$^{-1}$ (▼). Vehicle (□) group. All groups consisted of 8–12 animals. Groups treated with combination schedules exhibited significantly increased survival ($P < 0.01$) compared to those treated with cytotoxic drug alone the animals randomized into groups of 15–20 prior to treatment with various regimens 24 h later. XR9051 was administered i.p. (20 mg kg$^{-1}$) or p.o. (40 mg kg$^{-1}$) 1 h before and 3 h after doxorubicin i.v. (5 mg kg$^{-1}$). The animals were weighed twice weekly and on day 14 they were killed by cervical dislocation, the tumours excised and weighed. Student's *t*-test was used for statistical evaluation of the results.

### Human carcinoma xenografts

The ability of XR9051 to improve anti-tumour activity of several cytotoxic agents was evaluated using a number of human carcinoma xenografts in which MDR was mediated by P-gp expression.

### *2780AD ovarian carcinoma xenografts*

Efficacy studies in 2780AD ovarian carcinoma xenografts established in athymic mice were performed as described by Plumb

© Cancer Research Campaign 1999

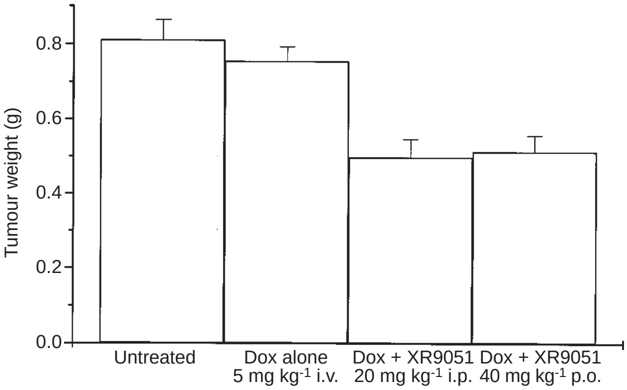

**Figure 2** Sensitization of doxorubicin anti-tumour effect on MC26 mouse colon carcinoma by XR9051 administered i.p. or p.o. Mice were implanted with MC26 tumour slurry s.c. and treated 24 h later. The modulator was administered i.p. or p.o. 1 h before and 3 h after doxorubicin i.v. (5 mg kg$^{-1}$) on day 1. The tumour weights were determined on day 14 after tumour implant. Values represent mean ± S.E.M., using 20 animals per group. Vehicle; vehicle + doxorubicin 5 mg kg$^{-1}$ i.v.; doxorubicin + XR9051 20 mg kg$^{-1}$ i.p. ($P < 0.001$); doxorubicin + XR9051 40 mg kg$^{-1}$ p.o. ($P < 0.001$). The *P*-values are in comparison with untreated animals or those treated with doxorubicin alone

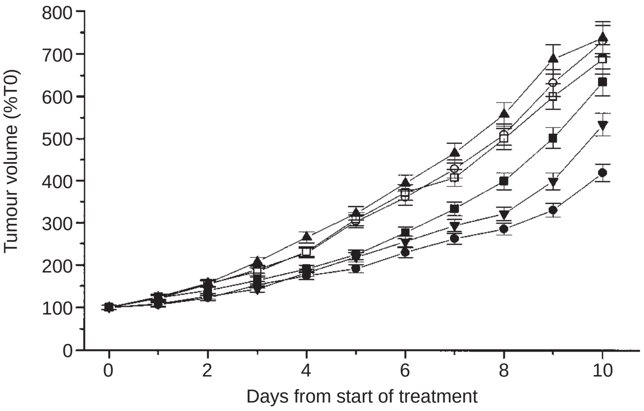

**Figure 3** Effect of XR9051 on activity of epirubicin in mice bearing 2780AD ovarian carcinoma xenografts. The modulator was administered i.p. 2 h before and 2 h after epirubicin i.v. on day 0. Vehicle (○), epirubicin 10 mg kg$^{-1}$ i.v. (□), XR9051 40 mg kg$^{-1}$ i.p. (▲), epirubicin + XR9051 i.p. at 20 mg kg$^{-1}$(■), 30 mg kg$^{-1}$ (▼) and 40 mg kg$^{-1}$ (●). Each group consisted of six animals. Tumour growth rate in animals treated with epirubicin + XR9051 at all doses was significantly different ($P < 0.01$) from those treated with drug alone or vehicle et al (1994). Briefly, female mice bearing s.c. 2780AD tumours with a mean diameter of 0.5–1.0 cm were randomized into groups of 6. The animals were treated (day 0) with combinations of XR9051 administered i.p. (20–40 mg kg$^{-1}$) with epirubicin i.v. (10 mg kg$^{-1}$). The modulator was administered 2 h before and after epirubicin. Both body weights and tumour diameters (two perpendicular values measured using callipers) were recorded at least twice a week. The tumour volume was estimated using spherical geometry (volume = $\pi/6 \times d^3$, where d is the mean diameter).

### *CH1/DOXr ovarian carcinoma xenografts*

CH1-sensitive and CH1/DOXr-resistant ovarian carcinoma cell lines, obtained from Dr Lloyd Kelland (Institute of Cancer Research, Sutton, UK), were also used for in vivo efficacy studies. The cells were maintained in Dulbecco's modified Eagle's medium (DMEM) supplemented with 10% FCS and 2 mM L-glutamine at 37°C in an atmosphere of 95% air–5% $CO_2$. The tumours were initially established s.c. by injection of $5 \times 10^6$ cells in athymic mice. The tumours were subsequently passaged s.c. by implantation of 1 mm$^3$ pieces on both flanks of female athymic mice aged 5–7 weeks. Mice bearing tumours with a mean diameter of 0.4–0.7 cm were randomized into groups (6–10 tumours) and treated with various regimens of modulator and cytotoxic agents (see Results section). Tumour volume and body weights were measured twice weekly post-treatment as outlined above.

### *H69/LX small cell lung carcinoma xenografts*

The efficacy of XR9051 was also evaluated using the H69/LX small cell lung carcinoma (SCLC) xenografts. The H69/LX and H69/LX4 cells were obtained from Dr Peter Twentyman (Imperial Cancer Research Fund, Cambridge, UK) and grown in vitro as outlined previously (Twentyman et al, 1986). The H69/LX cell xenografts were initially established by s.c. injection of $5 \times 10^6$ cells bilaterally in female athymic mice aged 5–7 weeks (ICR, Sutton). The established tumours were passaged by bilateral s.c. implantation of tumour tissue (1 mm$^3$) and were used for efficacy studies within ten passages. Animals bearing tumours with a mean diameter of 0.4–0.7 cm were randomized into groups of 8–12 tumours, and treated with various regimens of XR9051 in combination with doxorubicin and vincristine. Day 0 was the start of treatment. Tumour volume and body weights were measured twice weekly post-treatment as outlined above. Statistical significance was evaluated using the Mann–Whitney *U*-test (two tailed).

### Pharmacokinetic studies with XR9051

Female Balb/c mice (20–25 g) were injected i.v. with XR9051 (20 mg kg$^{-1}$) and at various times (5 min to 24 h) levels of the modulator were determined in plasma, liver, heart and brain in groups of three animals per time point. Blood was removed by cardiac puncture, after induction of anaesthesia, using heparinized syringes and centrifuged at 4°C to prepare plasma. The plasma and tissue samples were stored at –40°C until analysis by high pressure liquid chromatography (HPLC). Plasma levels of XR9051 at various times (30 min to 24 h) were also determined following a single oral dose of 50 mg kg$^{-1}$ in Balb/c mice (groups of three). In a separate experiment, levels of XR9051 in resistant H69/LX4 SCLC tumours were determined at various times following a single i.v. dose of 20 mg kg$^{-1}$ in tumour-bearing athymic mice.

The plasma samples at 4°C were extracted by addition of 3 volumes of –20°C methanol and incubation at –40°C for 15 min before centrifuging in a microfuge. The tissue samples were homogenized in 2–5 volumes (w/v) of PBS (pH 7.4) at 4°C and aliquots were extracted as described for plasma samples. The supernatants were analysed by HPLC as follows: the samples were eluted through an Inertsil ODS-2 column (250 × 4.6 mm) plus a guard column (10 × 2 mm) using a linear gradient of 60% solvent A + 40% solvent B to 100% solvent B. Solvent A and B consisted of 0.02 M triethylammonium acetate pH 4.1 and 80% acetonitrile in 0.02 M triethylammonium acetate pH 4.1, respectively. The flow rate was 1.0 ml min$^{-1}$ and XR9051 was detected at 327 nm. The concentration of XR9051

*© Cancer Research Campaign 1999*

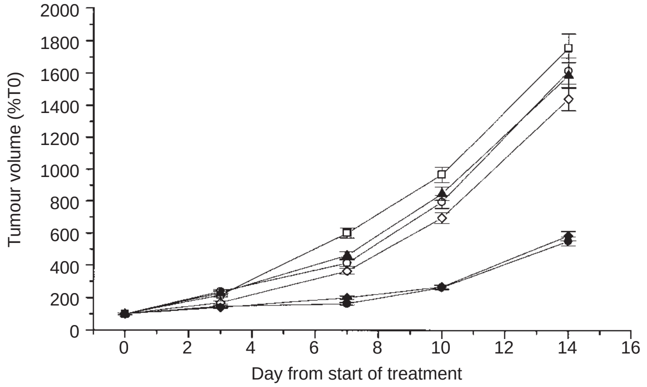

**Figure 4** Sensitization of Paclitaxel and etoposide anti-tumour effect on CHI DOXr ovarian carcinoma xenografts by XR9051 administered i.v. Animals bearing s.c. tumours were treated with 20 mg kg$^{-1}$ i.p. Paclitaxel alone (○) or Paclitaxel + XR9051 10 mg kg$^{-1}$ i.v. (●) on days 0, 2 and 4; or 30 mg kg$^{-1}$ i.p. etoposide alone (◇) or etoposide + XR9051 at 20 mg kg$^{-1}$ (◆) on days 0, 5 and 11; or 20 mg kg$^{-1}$ i.v. XR9051 alone (▲) or vehicle (□) on days 0, 2 and 4. Co-administration of XR9051 i.v. with Paclitaxel or etoposide significantly reduced tumour growth rate compared to either cytotoxic drug or vehicle alone (*P* < 0.01)

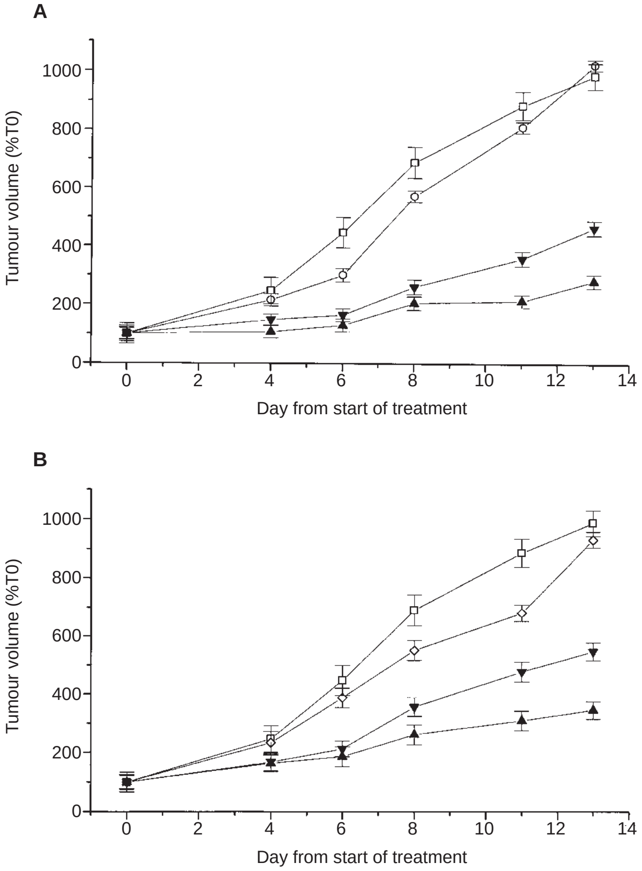

**Figure 5** (**A**) Effect of XR9051 on anti-tumour activity of vincristine in mice bearing H69/LX SCLC xenografts. Animals bearing s.c. tumours were treated with 0.5 mg kg$^{-1}$ vincristine i.p. (○) or XR9051 40 mg kg$^{-1}$ i.p. 1 h before vincristine (▼) or XR9051 40 mg kg$^{-1}$ p.o. 2 h before and 2 h after vincristine (▲); Vehicle (□) group. (**B**) Animals bearing s.c. tumours were treated with doxorubicin (4 mg kg$^{-1}$ i.p.) (◇) or XR9051 40 mg kg$^{-1}$ i.p. 1 h before doxorubicin (▼) or XR9051 40 mg kg$^{-1}$ p.o. 2 h before and 2 h after doxorubicin (▲); vehicle (□). Tumour growth rate was significantly (*P* < 0.01) reduced in animals treated with combination schedules compared with drug alone in the samples was determined from standard curves prepared using appropriate biological fluids. Pilot studies had shown that the recovery of added XR9051 from plasma was > 95%. The limit of quantitation in plasma samples was 50 ng ml$^{-1}$ and in tissue samples it was between 100 and 500 ng g$^{-1}$.

### RESULTS

### Efficacy in P388 murine leukaemia model

The sensitive and MDR P388 murine leukaemia cells implanted i.p. have been extensively used for evaluating the efficacy of MDR modulators (Tsuruo et al, 1981; Shinoda et al, 1989; Boesch et al, 1991; Hyafil et al, 1993). We have shown in previous studies that in vitro co-exposure to XR9051 fully restored sensitivity of the resistant cells (P388/DX Johnson) to several cytotoxic agents (Dale et al, 1998). The in vivo studies with P388/P-sensitive cells implanted i.p. in mice demonstrated that i.p. administration of vincristine (0.1 mg kg$^{-1}$) on days 1–5 or doxorubicin (2 mg kg$^{-1}$) on days 1, 4 and 7 after cell inoculation (day 0) significantly (*P* < 0.005) increased the lifespan of tumour-bearing animals compared to those treated with vehicle alone. Some drug-treated animals survived for between 50 and > 60 days, whereas vehicle or modulator alone treated animals survived for 12 days. Co-administration of XR9051 (20 or 40 mg kg$^{-1}$, i.v.) with either cytotoxic drug did not further increase the lifespan of the sensitive tumour-bearing animals. In contrast, treatment of P388/DX Johnson-resistant leukaemia-bearing mice with vincristine or doxorubicin in the absence of XR9051 had little or no effect on survival. However, co-administration of XR9051 significantly increased anti-tumour activity of both cytotoxic drugs in resistant tumour-bearing animals. The increase in lifespan of resistant tumour-bearing animals following treatment with combination schedules was not as great as that achieved with cytotoxic drugs alone in sensitive tumour-bearing animals. However, preliminary studies had shown that this difference may be due to the fact that the resistant P388/DX Johnson tumours were more aggressive and killed the host animals faster than the parental P388 tumours. The data suggested that the increase in survival time following identical cell kill would be greater in animals bearing parental than those bearing resistant tumours, and that the difference in survival time would increase as the number of cells killed increased. The initial efficacy studies in resistant leukaemia-bearing animals were performed following i.p. co-administration of XR9051 at 5, 10 and 20 mg kg$^{-1}$ with vincristine (0.1 mg kg$^{-1}$, i.p.) and demonstrated a dose-dependent increase in lifespan of resistant tumour-bearing animals (data not shown). Subsequent studies were performed with combination therapies in which XR9051 was administered at a site remote from the tumour (i.v. or p.o.), which is more clinically relevant. Again, significant dose-related increases in lifespan were observed when XR9051 was administered either i.v. or p.o. 1 h before vincristine on days 1–5, compared with cytotoxic drug alone (Figure 1A, B). Similar results were obtained following i.v. and p.o. administration of XR9051 with doxorubicin. Moreover, there was no significant increase in toxicity, as assessed by body weight loss, in the combination therapy groups (data not shown). An equivalent enhancement in anti-tumour activity of both cytotoxic drug was obtained

© Cancer Research Campaign 1999

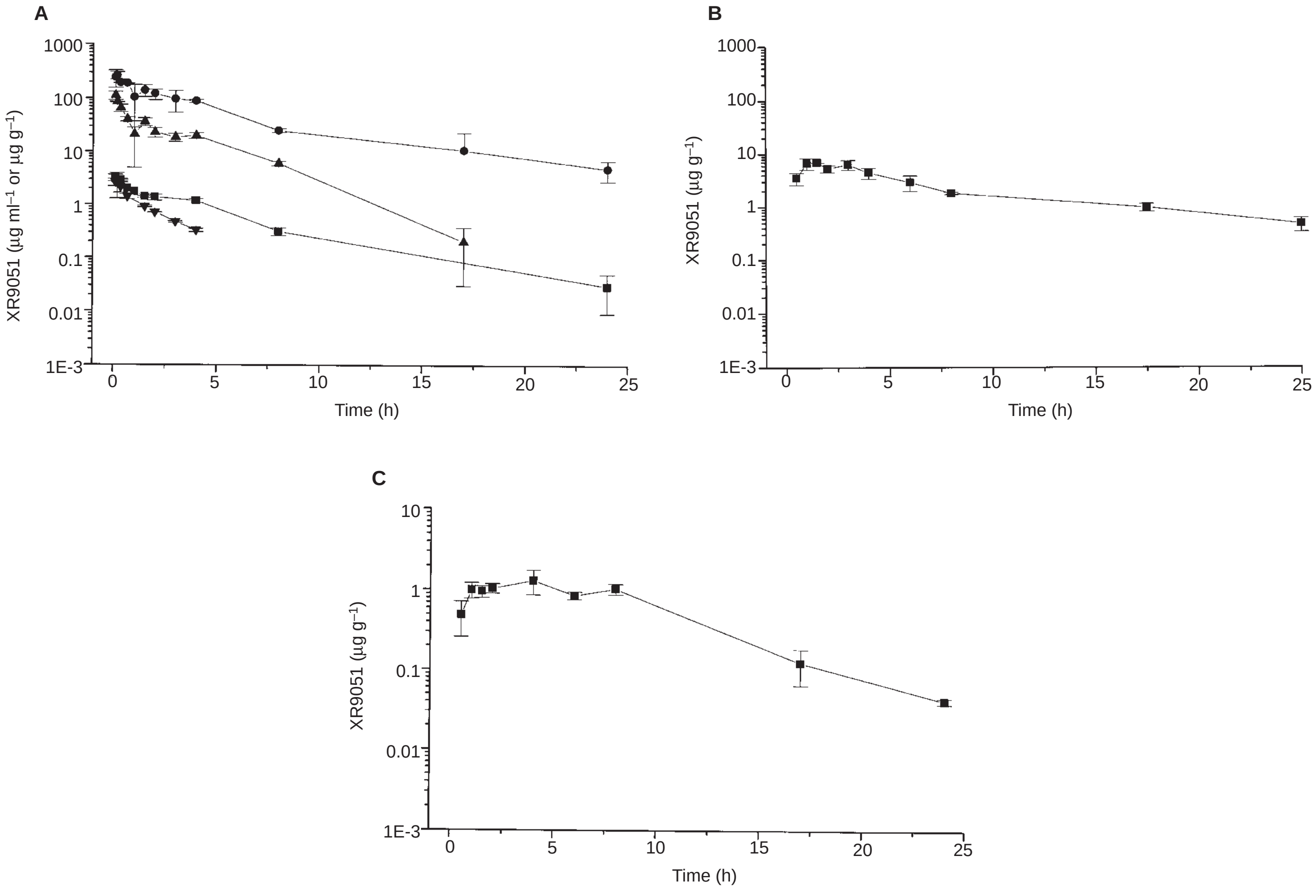

**Figure 6** (**A**) Concentration versus time curves obtained for XR9051 in plasma (■), liver (●), heart (▲) and brain (▼) after i.v. dose of 20 mg kg$^{-1}$ in Balb/c mice. (**B**) Levels of XR9051 in H69/LX4 multidrug-resistant human SCLC tumours after an i.v. dose of 20 mg kg$^{-1}$ in tumour-bearing nude mice. (**C**) Concentration versus time profile of XR9051 in plasma after p.o. administration of 50 mg kg$^{-1}$ in Balb/c mice. The values represent mean ± s.e.m of 3–5 animals per time point when the oral dose of XR9051 was two- to threefold greater than the i.v. dose. Administration of XR9051 alone by any route had no effect on survival of mice bearing sensitive or resistant tumours. These results are consistent with the in vivo modulatory activity being through inhibition of P-gp function.

### MC26 murine colon carcinoma studies

In vitro, 500 nM XR9051 enhanced the sensitivity of the intrinsically resistant MC26 cells to doxorubicin by three-fold (unpublished data). The ability of XR9051 to potentiate the anti-tumour activity of doxorubicin against s.c. MC26 tumours is shown in Figure 2. Treatment was started 24 h after tumour implantation. Administration of XR9051 either i.p. (20 mg kg$^{-1}$) or p.o. (40 mg kg$^{-1}$) at 0 and 4 h with doxorubicin (5 mg kg, i.v.) at 1 h resulted in significant ($P < 0.001$) inhibition of tumour growth on day 14 compared to cytotoxic drug alone. XR9051 alone had no significant inhibitory activity in pilot experiments. In addition, there were no differences in body weight gain between the various groups.

### Efficacy in human carcinoma xenografts

The ability of XR9051 to reverse P-gp-mediated MDR was evaluated in several human carcinoma xenografts grown s.c. in athymic mice. Figure 3 demonstrates that co-administration of XR9051 (i.p.) with epirubicin (10 mg kg$^{-1}$, i.v.) significantly reduced growth rate of MDR 2780AD ovarian carcinoma xenografts compared with either drug alone. Moreover, the potentiation of epirubicin anti-tumour activity was related to the dose of XR9051. The reduction in resistant tumour growth rate obtained by co-administration of 40 mg kg$^{-1}$ XR9051 was similar to that reported for the sensitive (2780) tumours following epirubicin treatment alone (Plumb et al, 1994), suggesting that full reversal of resistance was achieved with this combination therapy. The weight loss observed in groups treated with epirubicin plus XR9051 was similar to that observed in the group treated with cytotoxic drug alone (data not shown).

Studies in another MDR ovarian carcinoma (CH1/Doxr) xenograft showed that administration of Paclitaxel (20 mg kg$^{-1}$, i.p.) or etoposide (30 mg kg$^{-1}$, i.p.) with XR9051 i.v., 10 or 20 mg kg$^{-1}$, respectively (Figure 4) significantly reduced tumour growth rate compared with either drug alone and that the combination schedules were well-tolerated as judged by changes in body weights (data not shown). The reduction in growth rate of the resistant tumour was similar to that observed with the cytotoxic drug alone in the sensitive tumours (data not shown). Similarly, administration of XR9051 i.p. or p.o. significantly potentiated the anti-tumour activity of vincristine (0.5 mg kg$^{-1}$, i.p.) (Figure 5A) and doxorubicin (2 mg kg$^{-1}$) (Figure 5B) in H69/LX SCLC xenografts. Again the combination schedules were well-tolerated.

*© Cancer Research Campaign 1999*

### Pharmacokinetics of XR9051

The concentration time curves for XR9051 as determined in plasma, liver, heart and brain after a single i.v. dose of 20 mg $kg^{-1}$ are shown in Figure 6A. Using a two-compartment model the apparent elimination half-life for XR9051 in plasma, liver, heart and brain was 3.96, 4.93, 2.00 and 1.15 h respectively. The steady-state volume of distribution for XR9051 was 9.6 l $kg^{-1}$ and the high concentrations of the compound in tissues are consistent with this large volume of distribution. The area under the concentration time curves (AUC) from time 0–∞ for plasma was 11.9 μg. h $ml^{-1}$ The ratio between AUC for tissue:plasma for liver, heart and brain were 79.6, 16.9 and 0.3 respectively. The intravenous pharmacokinetic parameters were linear between 20 and 100 mg $kg^{-1}$ doses (data not shown). In a separate experiment, in H69/LX4 SCLC xenograft-bearing athymic mice, a maximum concentration of 6.94 μg $g^{-1}$ (9.4 μM) of XR9051 in tumour tissue was observed 1.5 h after a single i.v. dose of 20 mg $kg^{-1}$ (Figure 6B). This concentration was well above that observed in plasma at the same time. The apparent elimination half-life of the compound in tumour tissue was 8.79 h and the $AUC_{0-\infty}$ was 57.1 μg. h $ml^{-1}$. Following oral administration of 50 mg $kg^{-1}$ XR9051 $C_{max}$ in plasma was 1.3 μg $ml^{-1}$ (1.8 μM) and $T_{max}$ was estimated to be 3.4 h (Figure 6C). The $AUC_{0-24\ h}$ was 13.5 μg. h $ml^{-1}$ and the oral bioavailability of XR9051 was estimated to be about 46%.

### DISCUSSION

Although a number of first-generation compounds have been reported to reverse P-gp-mediated multidrug resistance in vitro, few have shown beneficial effects in vivo in MDR tumour-bearing animals due to lack of potency. The present in vivo studies with XR9051 have shown that this modulator is potent and well-tolerated at effective doses. These results are in agreement with the in vitro results (Dale et al, 1998). Co-administration of XR9051 has shown clear dose-dependent enhancement of anti-tumour activity of five cytotoxic agents (vincristine, doxorubicin, epirubicin, Paclitaxel and etoposide) in mice bearing both murine and human tumour xenografts exhibiting multidrug resistance. Moreover, efficacy has been observed following both parenteral (i.p. and i.v.) and oral routes of administration of modulator.

In the P388/MDR tumour-bearing animals the anti-tumour activity of vincristine and doxorubicin was significantly increased in a dose-dependent manner by i.v. (between 10 and 40 mg $kg^{-1}$) and oral (60–120 mg $kg^{-1}$) co-administration of XR9051. In contrast, cyclosporin A (CsA) at its maximum tolerated oral dose in combination with vincristine did not enhance the activity of this drug in these animals (data not shown). Boesch et al (1991) have also reported no enhancement of vinblastine activity by co-administration of CsA orally in MDR P388 tumour-bearing animals. However, CsA administered i.p. was reported to increase vincristine anti-tumour activity in animals inoculated with P388 cells that had been transfected with the human MDR1 gene (Yang et al, 1994). The resistance factor of this cell line to vincristine was low (22-fold) compared to the cell line used in our studies (70-fold). Several second-generation MDR modulators have been reported to exhibit in vivo efficacy in murine tumour models including MS-209 (Sato et al, 1995), S9788 (Cros et al, 1992), the non-immunosuppressive cyclosporine A analogue, PSC 833, which showed significant oral activity (Boesch et al, 1991), GG918 (Hyafil et al, 1993) and LY335979 (Dantzig et al, 1996). However, direct comparison of the potency between modulators is difficult due to differences in tumour growth rates, treatment schedules and experimental design.

In addition to the murine tumour models, XR9051 showed significant modulatory activity in mice bearing MDR human ovarian (2780AD and CH1/DOXr) and SCLC (H69/LX) xenografts. Either complete or near complete reversal of resistance to vincristine, doxorubicin, epirubicin, Paclitaxel and etoposide was achieved in these models by co-administration of XR9051 either parenterally or orally at doses that were well-tolerated (acute toxicity studies with XR9051 alone in mice showed no lethality following 75 mg $kg^{-1}$ i.v. and 2000 mg $kg^{-1}$ p.o. dose). Moreover, the combination therapy schedules were well-tolerated. The oral potency of XR9051 was two- to three-fold less than that observed following i.v. administration in these and the murine tumour models. This correlates well with the oral bioavailability of XR9051.

The in vivo potency of XR9051 correlates well with the pharmacokinetic profile of the modulator, which shows that, following i.v. administration, the compound rapidly distributes and accumulates in tissues and has a long elimination half-life. In fact, over a 25 h period after a single i.v. dose of 20 mg $kg^{-1}$ XR9051, concentrations in H69/LX4 MDR tumours (range 0.6–9.4 μM) remain above levels required for full reversal of resistance in vitro (300–500 nM). The in vivo efficacy of MDR modulators may be due indirectly through an increase in pharmacokinetics of the cytotoxic agents as a result of reduced elimination or metabolism (Wacher et al, 1995; Colombo et al, 1996). Indeed, such changes are thought to be at least partially responsible for efficacy of some modulators, such as CsA and PSC 833, but not others such as GG918 and S9788 (Hyafil et al, 1993; Colombo et al, 1996). We are currently determining the effects of XR9051 on pharmacokinetics of cytotoxic agents and these studies will be reported separately.

In conclusion, these results show that XR9051 is a potent and specific modulator of P-gp-dependent MDR in vivo. It restores sensitivity of syngeneic and human tumour xenografts to a range of cytotoxic agents at doses that are well-tolerated. The data reported here fully support our previous results demonstrating that XR9051 (300–500 nM) fully sensitized a panel of resistant cells to a broad range of cytotoxic drugs (Dale et al, 1998). Furthermore, the activity of XR9051 persisted in vitro for an extended period following removal from the culture medium. This feature should give the modulator a significant advantage for clinical administration. Thus the in vitro and in vivo activity of XR9051 together with its pharmacokinetic profile suggest that XR9051 may significantly improve treatment of MDR cancers.

### ACKNOWLEDGEMENTS

We thank Karen Flatman, Andrew Silverthorne, Christopher Barnes, Rachael Johnson and Teresa Morris for their technical support.

### REFERENCES

Boesch D, Gaveriaux C, Jachez B, Pourtier-Manzanedo A, Bollinger P and Loor F (1991) In vivo circumvention of P-glycoprotein-mediated multidrug resistance of tumour cells with SDZ PSC 833. *Cancer Res* **51**: 4226–4233

Childs S and Ling V (1994) The MDR superfamily of genes and its biological implications. In *Important Advances in Oncology*, De Vita VT, Hellman S and Rosenberg SA (eds), pp. 21–36. Lippincott Co: Philadelphia

© Cancer Research Campaign 1999

Colombo T, Paz OG and D'Incalci M (1996) Distribution and activity of doxorubicin combined with SDZ PSC 833 in mice with P388 and P388/DOX leukaemia. *Br J Cancer* **73**: 866–871

Cros S, Guilbaud N, Berlion M, Dunn T, Regnier G, Dhainaut A, Atassi G and Bizzari JP (1992) In vivo evidence of complete circumvention of vincristine resistance by a new triazinoaminopiperidine derivative S9788 in P388/VCR leukaemia model. *Cancer Chemother Pharmacol* **30**: 491–494

Dale IL, Tuffley W, Callaghan R, Holmes JA, Martin K, Luscombe M, Mistry P, Ryder H, Stewart AJ, Charlton P, Twentyman PR and Bevan P (1998) Reversal of P-glycoprotein (P-gp)-mediated multidrug resistance by XR9051, a novel diketopiperazine derivative. *Br J Cancer* **78**: 885–892

Dalton WS (1994) Is p-glycoprotein a potential target for reversing clinical drug resistance? *Curr Opin Oncol* **6**: 595–600

Dantzig AH, Shepard RL, Cao J, Law KL, Ehlhardt WJ, Baughman TM, Bumol TF and Starling JJ (1996) Reversal of P-glycoprotein-mediated multidrug resistance by a potent cyclopropyldibenzosuberane modulator, LY335979. *Cancer Res* **56**: 4171–4179

Ferry DR, Traunecker H and Kerr DJ (1996) Clinical trials of P-glycoprotein reversal in solid tumours. *Eur J Cancer* **32A**: 1070–1081

Germann UA (1996) P-glycoprotein: a mediator of multidrug resistance in tumour cells. *Eur J Cancer* **32A**: 927–944

Gottesman MM and Pastan I (1993) Biochemistry of multidrug resistance mediated by the multidrug transporter. *Ann Rev Biochem* **62**: 385–427

Hyafil F, Vergely C, Du Vignaud P and Grand-Perret T (1993) In vitro and in vivo reversal of multidrug resistance by GF120918, an acridonecarboxamide derivative. *Cancer Res* **53**: 4595–4602

Johnson RK, Chitnis MP, Embrey WM and Gregory EB (1978) In vivo characteristics of resistance and cross-resistance of an adriamycin-resistant subline of P388 leukaemia. *Cancer Treat Rep* **62**: 1535–1547

Lum BL, Fisher GA, Brophy NA, Yahanda AM, Adler KM, Kaubisch S, Halsey J and Sikic BI (1993) Clinical trials of modulation of multidrug resistance. Pharmacokinetic and pharmacodynamic considerations. *Cancer* **72**: 3502–3514

Plumb JA, Wishart GC, Setanoians A, Morrison JG, Hamilton TN, Bicknell SR and Kaye SB (1994) Identification of a multidrug resistance modulator with clinical potential by analysis of synergistic activity in vitro, toxicity in vivo and growth delay in a solid human tumour xenograft. *Biochem Pharmacol* **47**: 257–266

Sato W, Fukazawa N, Nakanishi O, Baba M, Suzuki T, Yano O, Naito M and Tsuruo T (1995) Reversal of multidrug resistance by a novel quinoline derivative, MS-209. *Cancer Chemother Pharmacol* **35**: 271–277

Shinoda H, Inaba M and Tsuruo T (1989) In vivo circumvention of vincristine resistance in mice with P388 leukaemia using a novel compound AHC-52. *Cancer Res* **49**: 1722–1726

Spoelstra EC, Dekker H, Schuurhuis GJ, Broxterman HJ and Lankelma J (1991) P-glycoprotein efflux pump involved in the mechanism of intrinsic drug resistance in various colon cancer cell lines. Evidence for a saturation of active daunorubicin transport. *Biochem Pharmacol* **41**: 349–359

Tsuruo T, Iida H, Tsukagoshi S and Sakurai Y (1981) Overcoming of vincristine resistance in P388 leukaemia in vivo and in vitro through enhanced cytotoxicity of vincristine and vinblastine by verapamil. *Cancer Res* **41**: 1967–1972

Twentyman PR, Fox NE, Wright KA and Bleehen NM (1986) Derivation and preliminary characterisation of adriamycin resistant lines of human lung cancer cells. *Br J Cancer* **53**: 529–537

Wacher VJ, Wu CY and Benet LZ (1995) Overlapping substrate specificities and tissue distribution of cytochrome P450 3A and P-glycoprotein: implications for drug delivery and activity in cancer chemotherapy. *Mol Carcinog* **13**: 129–134

Yang J-M, Goldenberg S, Gottesman MM and Hait WN (1994) Characteristics of P388/VMDRC.04, a simple, sensitive model for studying P-glycoprotein antagonists. *Cancer Res* **54**: 730–737

 *© Cancer Research Campaign 1999*
